# Recent Advances in the Bacterial Flagellar Motor Study

**DOI:** 10.3390/biom11050741

**Published:** 2021-05-17

**Authors:** Tohru Minamino, Keiichi Namba

**Affiliations:** 1 Graduate School of Frontier Biosciences, Osaka University, 1-3 Yamadaoka, Suita, Osaka 565-0871, Japan; 2 RIKEN SPring-8 Center and Center for Biosystems Dynamics Research, 1-3 Yamadaoka, Suita, Osaka 565-0871, Japan; 3 JEOL YOKOGUSHI Research Alliance Laboratories, Osaka University, 1-3 Yamadaoka, Suita, Osaka 565-0871, Japan

The bacterial flagellum is a supramolecular motility machine that allows bacterial cells to swim in liquid environments. The flagellum is composed of the basal body, which acts as a rotary motor, the filament, which functions as a helical propeller, and the hook, which connects the basal body and filament and works as a universal joint to smoothly transmit torque produced by the motor to the filament. The flagellar motor is composed of a rotor ring complex and multiple transmembrane stator units, each of which acts as an ion channel to couple the ion flow through the channel to torque generation. The flagellar motor is placed under the control of sensory signal transduction networks, thereby allowing bacterial cells to migrate towards more desirable environments for their survival. The entire structure of the flagellum and flagellar component proteins are highly conserved among bacterial species. However, novel and divergent structures associated with the flagellar motor are clearly observed by in situ structural analyses of flagellar motors derived from different bacterial species [1,2,3].

The scope of this Special Issue is to cover recent advances in our understanding of the structures and functions of the bacterial flagellar motor derived from different bacterial species. This Special Issue includes ten review articles [4,5,6,7,8,9,10,11,12,13] and eleven original research papers [14,15,16,17,18,19,20,21,22,23,24] from well-known experts in the field.

All review articles provide both expert and non-expert readers with advances in understanding the structures and functions of the bacterial flagellum. They highlight the most recent observations and illustrate perspectives for future research [4,5,6,7,8,9,10,11,12,13].

The amino acid sequence of the distal rod protein FlgG is very similar to that of the hook protein FlgE. The FlgG rod structure is straight and rigid, whereas the hook adopts a curved form with high bending flexibility. Saijo-Hamano et al. solved a crystal structure of the FlgG fragment missing both N- and C-terminal disordered regions and fitted the atomic model of the FlgG fragment into a density map of the FlgG rod by electron cryomicroscopy (cryoEM). They found that an N-terminal short segment called L-stretch stabilizes intermolecular packing interactions, making the rod straight and rigid. As a result, the rod functions as a drive shaft of the flagellar motor [14]. Horváth and Kato et al. carried out cryoEM image analysis of the straight polyhook structure and provided structural evidence that domain Dc of FlgE with a long β-hairpin structure connecting domains D0 and D1 not only contributes to the structural stability of the hook but also allows the bending flexibility of the hook so that the hook can function as a universal joint [15].

*Salmonella enterica* has two distinct flagellin genes, namely *fliC* and *fljB*, on the genome and autonomously switches their expression at a frequency of 10^−3^–10^−4^ per cell per generation. Yamaguchi et al. carried out functional and structural analyses of the filaments formed by either FliC or FljB and provided evidence that domain D3 of flagellin molecules plays an important role not only in changing the antigenicity of the filament but also in optimizing the motility function of the filament as a propeller under different environmental conditions [16].

To construct the flagellum on the bacterial cell surface, the flagellar type III secretion system (fT3SS) transports flagellar building blocks from the cytoplasm to the distal end of the growing flagellar structure. Terashima et al. developed in vitro protein transport assays using inverted membrane vesicles and provided direct evidence that coordinated flagellar protein export and assembly can occur at the post-translational level [17].

A non-flagellated bacterium *Lysobacter enzymogenes* OH11 moves on solid surfaces using type IV pili. Interestingly, this bacterium encodes highly homologous fT3SS genes on its genome. Fulano et al. constructed fT3SS-knockout mutant strains and provided evidence that some fT3SS components are required for the twitching motility of *L. enzymogenes*. Thus, the homologous components of the fT3SS seem to have acquired a divergent function that controls the twitching motility [18].

MotA and MotB form a transmembrane proton channel complex to couple the proton flow through the channel with torque generation. The MotAB stator complex autonomously controls its proton channel activity in response to changes in the environment. Morimoto et al. provided experimental evidence that the N-terminal cytoplasmic tail of MotB regulates the gating of the MotAB proton channel [19]. Furthermore, Naganawa and Ito provided an interesting clue of how the stator unit selects the coupling ion to drive flagellar motor rotation [20].

Onoe et al. showed that the *Paenibacillus* MotAB complex, which was originally thought to conduct divalent cations such as Ca^2+^ and Mg^2+^ to drive flagellar motor rotation, can work as a stator unit in the *E. coli* flagellar motor and that this stator unit directly converts the energy released from the proton influx to motor rotation in *E. coli* [21].

The chemotaxis signaling protein, namely CheY-P, binds to a rotor of the flagellar motor to switch its rotational direction from counterclockwise to clockwise in a highly cooperative manner. The cytoplasmic level of CheY-P largely fluctuates so that *E. coli* cells respond to changes in the environment rapidly and efficiently to migrate toward more desirable conditions. Che et al. analyzed the coordination of directional switching between flagellar motors on the same cell and provided evidence suggesting that the fluctuation of the cytoplasmic CheY-P level coordinates rotation among flagellar motors and regulates steady-state run-and-tumble swimming of cells to facilitate efficient responses to environmental changes [22].

A motile *Methylobacterium* ME121 strain is more motile when they grow together with a non-motile *Kaistia* 32K strain. Usui et al. purified a swimming acceleration factor from the culture supernatant and found that extracellular polysaccharides, which they named the K factor, facilitate the flagellar motor function of the ME121 strain [23].

Lysophosphatidic acid acyltransferase (LPAAT) introduces fatty acyl groups into the sn-2 position of membrane phospholipids. *E. coli* has another LPAAT homolog named YihG in addition to PlsC, which is essential for the growth of *E. coli*. Toyotake et al. constructed a *yihG* null mutant (∆*yihG*) and provided evidence suggesting that YihG has specific functions related to flagellar assembly through the modulation of the fatty acyl composition of membrane phospholipids [24].

Thus, the studies included in this Special Issue illustrate various examples of the recent progress in the studies on the conserved structure and function of the flagellar motor as well as its structural and functional diversities among different bacterial species.

Finally, we would like to thank all authors for their great contributions to this Special Issue and Fumiaki Makino and Tomoko Yamaguchi for creating the cover image.
